# Imaging of Left Main Coronary Artery; Untangling the Gordian Knot

**DOI:** 10.31083/j.rcm2401026

**Published:** 2023-01-12

**Authors:** Anastasios Apostolos, Andreas Gerakaris, Evropi Tsoni, Konstantinos Pappelis, Georgios Vasilagkos, Elena Bousoula, Athanasios Moulias, Konstantinos Konstantinou, Kyriakos Dimitriadis, Grigoris V. Karamasis, Adel Aminian, Konstantinos Toutouzas, Periklis Davlouros, Grigorios Tsigkas

**Affiliations:** ^1^Department of Cardiology, University Hospital of Patras, 26504 Patras, Greece; ^2^First Department of Cardiology, Medical School, National and Kapodistrian University of Athens, Hippokration Hospital, 11527 Athens, Greece; ^3^Department of Ophthalmology, University Medical Center Groningen, University of Groningen, 9700 Groningen, The Netherlands; ^4^Cardiology Department, Tzaneio Hospital, 18536 Pireaus, Greece; ^5^Second Department of Cardiology, Medical School, National and Kapodistrian University of Athens, Attikon University Hospital, 12462 Athens, Greece; ^6^Department of Cardiology, Centre Hospitalier Universitaire de Charleroi, 6042 Charleroi, Belgium

**Keywords:** left main disease, left main coronary artery, invasive coronary angiography, intravascular ultrasound, IVUS, optical coherence tomography, OCT, percutaneous coronary intervention, PCI

## Abstract

Left Main Coronary Artery (LMCA) disease is considered a standout manifestation of coronary artery disease (CAD), because it is accompanied by the highest mortality. Increased mortality is expected, because LMCA is responsible for supplying up to 80% of total blood flow to the left ventricle in a right-dominant coronary system. Due to the significant progress of biomedical technology, the modern drug-eluting stents have remarkably improved the prognosis of patients with LMCA disease treated invasively. In fact, numerous randomized trials provided similar results in one- and five-year survival of patients treated with percutaneous coronary interventions (PCI) -guided with optimal imaging and coronary artery bypass surgery (CABG). However, interventional treatment requires optimal imaging of the LMCA disease, such as intravascular ultrasound (IVUS) and optical coherence tomography (OCT). The aim of this manuscript is to review the main pathophysiological characteristics, to present the imaging techniques of LMCA, and, last, to discuss the future directions in the depiction of LMCA disease.

## 1. Introduction

Cardiovascular disease (CVD) remains the leading cause of death globally, responsible for approximately 17.9 million deaths in 2016 [1, 2, 3, 4]. Coronary artery disease (CAD), presented either as an acute coronary syndrome (ACS) or chronic coronary syndrome (CCS), is the main manifestation of CVD, affecting the majority of cardiac patients and being the operative event for most heart diseases [5, 6, 7, 8].

Left Main Coronary Artery (LMCA) disease is considered a standout manifestation of CAD, because it is accompanied by the highest mortality. When left untreated, the three-year mortality is estimated at 63%, which is considerably higher than in coronary lesions located in other segments of the coronary artery tree [9]. Taking into consideration the anatomy and physiology of the coronary circulation, increased mortality is expected, because LMCA is responsible for supplying up to 80% of total blood flow to the left ventricle (LV) in a right-dominant coronary system [10]. Thus, occlusion of the LMCA puts a significant portion of the myocardium under high risk. LMCA disease is a frequent finding in invasive coronary angiography (ICA). It is estimated that about 4% of ICA examinations reveal LMCA disease [11]. In addition, ICA reveals LMCA stenosis in about 5% and 7% of patients with stable angina and acute syndrome, respectively [12]. About 5–10% of these patients present isolated LMCA [13].

The non-pharmaceutical treatment of LMCA disease has contributed to reduction in morbidity and mortality. Until recently, surgery was considered the gold standard approach for patients suffering from LMCA disease. Nowadays, both surgical and interventional revascularization have achieved comparable results in long-term follow-up; thus, a personalized approach is required for selecting the optimal therapeutic strategy [14, 15, 16].

The aim of this manuscript is to review the main pathophysiological characteristics, to present the imaging techniques of LMCA and, last, to shed light on the future directions in the depiction of this disease.

## 2. Anatomical and Pathophysiological Characteristics of LMCA Disease

Typically, the LMCA arises from the aorta, below the sinotubular junction and especially from the left sinus of Valsalva. It runs between the pulmonary trunk and the left atrial appendage and ends up bifurcating into two major branches: the Left Anterior Descending (LAD) and the Left Circumflex (LCx) artery [17]. A third branch, known as the intermediate ramus, arises from the LMCA in 30% of the general population [17]. The LMCA can be divided in three areas: the ostium, the trunk or shaft, and the distal vessel. The shaft and distal vessel present similar morphology; similar to epicardial vessels, they are composed of three layers (adventitia, median, and intima). Nevertheless, the ostium lacks an adventitia layer and presents more elasticity, compared to other coronary vessels [18]. The total length of the LMCA is estimated at about 10.5 ± 5.3 mm, while the mean diameter is estimated at 3.9 ± 0.4 mm and 4.5 ± 0.5 mm in women and men, respectively [19]. Moreover, cases have been described in which the LMCA does not exist and the LAD and LCx have separate orifices; the prevalence of this anatomical variation is estimated between 0.2% and 1.6% [20, 21].

Interestingly, the composition of the atherosclerotic plaque of the LMCA differs significantly from lesions in other segments of the coronary artery tree. Specifically, LMCA plaques are characterized by minimal necrotic core content and thicker cap fibroatheroma [22, 23]. Regarding the plaque distribution, atherosclerotic plaques develop more frequently in segments with lower shear stress; thus, the most common locations of these lesions are the lateral walls of the bifurcation to the LAD and LCx [24]. Atherosclerotic lesions rarely appear on the carina of the bifurcation, probably due to the high shear stress at this location [25]. Due to the above hydrological phenomena, thrombus formation in LMCA could be rarely observed. Nevertheless, it could be observed in special situations, such as cocaine use [26]. The vast majority of plaques are located in the distal part of the LMCA and are frequently extended to the proximal LAD, while the ostium is rarely implicated [27, 28]. However, lesions appear mainly near the ostium and not at the bifurcation in the short LMCA (<10 mm), probably due to the high shear stress and rheological laws [29]. The site of plaques has significant prognostic role, because percutaneous coronary interventions (PCI) in lesions of the distal LMCA is technically more demanding and with poorer outcomes [30]. When LCx imaging is suboptimal and ostia disease exists, intravascular ultrasound (IVUS) or optical coherence tomography (OCT) are useful for choosing the optimal bifurcation strategy: an upfront two-stent or provisional stenting strategy [31].

## 3. Imaging Modalities for LMCA Disease Depiction

Current guidelines support PCI as an alternative and equivalent treatment to surgical revascularization in patients with LMCA disease and low or intermediate (SYNTAX Synergy Between PCI With Taxus and coronary artery bypass surgery [CABG]) score [32]. These patients are unsuitable for surgery or present less complex disease. Patients with high (>32) SYNTAX score should be treated surgically. Intravascular imaging is considered mandatory before LMCA stenting, for the achievement of optimal results [32].

Imaging of LMCA stenosis is considered critical for its optimal evaluation and ideal management. As a result of progress in interventional cardiology, there exist both invasive and noninvasive methods to this end. Invasive assessment includes OCT and IVUS, whereas noninvasive includes mostly coronary computed tomography angiography (CCTA) [33]. Each imaging modality provides both advantages and disadvantages, which are analyzed below (Graphical abstract figure).

ICA remains the gold standard and the first-line diagnostic tool used in LMCA disease. Currently, transradial and distal transradial transluminal angiography is a safe and fast procedure, providing the cardiologist with the opportunity to perform all required interventions [34, 35]. Historically, an angiographic diameter stenosis of more than 50% of the LMCA lumen has been established as a cutoff limit for distinguishing significant disease. Patients with stenosis greater than 50% of the lumen’s diameter should be treated invasively or surgically. However, the degree of stenosis plays a pivotal role in the prognosis of such patients. Numerous studies have supported that patients with an estimated stenosis between 50 and 70% have significantly higher survival than those with stenosis exceeding 70% [36].

Nevertheless, the interpretation of angiographic views of the LMCA is frequently a challenge for invasive cardiologists. Several issues, such as overlap of branches, eccentric plaques, two-dimensional imaging, foreshortening of arteries, catheter displacement, and angle view of the LMCA, could lead to misinterpretation of disease severity. Moreover, angiographic evaluation remains subjective and may differ among operators. Generally, pathological studies support that ICA underestimates the extent of LMCA disease.

Taking the above into consideration, ICA remains the first step in the invasive assessment of LMCA disease, but is inadequate alone; thus, other techniques should accompany it for more accurate evaluation of the lesions, especially in patients with moderate stenosis (40–70%).

### 3.1 Coronary Computed Tomography Angiography (CCTA)

CCTA is the only noninvasive modality used in LMCA imaging. The examination is identical to conventional computed tomography; nevertheless, it is synchronized with electrocardiogram and special software is required for image processing [37]. Generally, CCTA provides high negative predictive value and it can confidently rule out obstructive CAD. Consequently, the necessity for ICA is significantly reduced. For patients with LMCA disease, CCTA provided an accuracy of 97.4% for the detection of CAD [38]. Dharampal *et al*. [39] supported that CCTA accurately detected and excluded left main and/or three-vessel CAD. Moreover, they estimated that the sensitivity, specificity, positive, and negative predictive value were 95%, 83%, 53%, and 99%, respectively. However, CCTA overestimates high-risk CAD in 47% of the patients [39]. CCTA played a crucial role in the recent ISCHEMIA trial, as it was used for excluding patients with LMCA disease. Indeed, its diagnostic ability was confirmed, as ICA revealed LMCA stenosis of more than 50% in only 2.9% patients without LMCA disease, according to CCTA [40]. In addition, CCTA could be used in patients with anomalous LMCA origin, in patients suffering from catheter-induced vasospasm, and in those having undergone coronary artery bypass surgery [41, 42, 43]. Last, CCTA has been proven as an acceptable solution for the detection of in-stent stenosis in LMCA. Although it remains inferior to ICA, it could be a safe and fast solution for the evaluation of patients treated with PCI [44, 45].

However, CCTA presents several limitations that should be addressed. First, it tends to overestimate stenosis severity, compared to ICA. Second, it is frequently affected by motion artefacts, caused by cardiac or breathing motion. As a result, CCTA should be avoided in patients with extensive coronary calcification, irregular heart rate, significant obesity, and inability to cooperate [14].

### 3.2 Intravascular Ultrasound (IVUS)

With over two decades of conventional use in LMCA disease evaluation, grayscale IVUS represents the mainstay of the LMCA intermediate lesions assessment [46]. A small transducer is mounted at the tip of a flexible catheter, emitting ultrasound in the 10 to 60 MHz range, utilizing ultrasonography and acoustic properties for tissue characterization. Two types of IVUS catheters are used in current clinical practice; the mechanical and the phased array. The former has a single mechanical head placed on the tip, which rotates to visualize the coronary artery cross-sectionally [47]. Generally, image quality seems to be superior using the mechanical transducer, with an overall resolution estimated between 100 and 150 micrometers. Newer-generation devices provided higher frequency and, as a result, improved the resolution. However, the higher the frequency, the poorer the penetration and the more increased the reflectivity of blood, which limit its clinical applications. Phased array catheters are equipped with multiple transducers, which are fixed in specific positions. Each transducer acts as a single unit; all signals are collected and then the IVUS image is created. This modality requires more sophisticated and advanced technology, in order to produce a sufficient optical result.

Different measurements can be obtained by the IVUS, but the clinically relevant measurement of IVUS is the minimum luminal area (MLA). MLA has been studied extensively and can accurately predict whether revascularization is required or can be avoided [48]. Initial reports demonstrated that MLA less than 9 ${\mathrm{mm}}^{2}$ or lumen stenosis greater than 50% constitute a hemodynamically significant stenosis. Fassa and colleagues decreased the lower limit of MLA to 7.5 ${\mathrm{mm}}^{2}$ and major cardiovascular events (MACE) rates in patients treated invasively and pharmaceutically did not show any differences in three years of follow-up, using this threshold [49]. Numerous trials have studied different MLA thresholds with comparable results, presented in Table 1 (Ref. [49, 50, 51, 52, 53]). Currents guidelines have set the cutoff at 6.0 ${\mathrm{mm}}^{2}$, which could be applied globally [50]. In the recent EXCEL trial, which compared PCI with CABG for LMCA disease, this value was used as the cutoff in MLA [54]. Smaller MLA thresholds have been studied in specific populations and larger trials are required for validation of their results. According to the recent European position paper on intravascular imaging, LMCA IVUS-derived MLA >6 ${\mathrm{mm}}^{2}$ could be safely deemed non-ischemic, <4.5 ${\mathrm{mm}}^{2}$ should be regarded as ischemia-generating, and the intermediate values are considered as ‘grey-zone’, thus further assessment of ischemia should be performed [55].

**Table 1.** **Studies comparing the optimal MLA threshold**.

First author	Year of publication	Number of patients	MLA threshold
Jasti *et al*. [52]	2004	55	≤5.9 ${\mathrm{mm}}^{2}$
Fassa *et al*. [49]	2005	214	<7.5 ${\mathrm{mm}}^{2}$
de la Torre Hernandez *et al*. [50]	2011	354	<6 ${\mathrm{mm}}^{2}$
Kang *et al*. [51]	2011	403	<4.8 ${\mathrm{mm}}^{2}$
Park *et al*. [53]	2014	112	<4.5 ${\mathrm{mm}}^{2}$

MLA, Minimum Lumen Area.

Another feature of LMCA lesions is the existence of calcification, which is systematically underestimated in ICA. The identification and quantification of calcium is crucial because its presence is associated with poorer prognosis and suboptimal stent placement. Significant calcification could drive the carina to shift toward the LCx; thus, the kissing-balloon technique should be performed [56]. Indeed, calcium allocation affects the therapeutic algorithm; when the calcific arch surpasses 180°, a dedicated plaque modification strategy of calcified lesions is suggested. Rotational atherectomy remains a reliable approach for pre-treatment of heavily calcified lesions, with acceptable in-hospital results [57]. Intravascular lithotripsy could be also considered for lesion preparation in calcific distal LMCA disease [58, 59, 60, 61]. When extensive calcification or high plaque load is located in bifurcations, the optimal technique is kissing balloons. In the remaining cases, pre-dilation with noncompliant balloons could be considered a sufficient treatment choice [62].

In addition to the assessment of LMCA disease, IVUS is used for PCI guidance, before and after stent implantation. Prior to PCI, the operators should use IVUS, in order to characterize the plaque composition and distribution, to select the suitable stent length and size, and, last, to consider whether alternative interventions (lithotripsy or atherectomy) should be applied. After the stent’s placement, IVUS should be performed to optimize the end result by assessing the plaque coverage and sufficient stent expansion [63].

Stent underexpansion has been demonstrated as the main risk factor for stent thrombosis and target lesion failure [64]. Moreover, suboptimal stent expansion has been correlated with hard endpoints, as it has been established as a serious prognostic factor for MACEs in 403 patients (adjusted. Hazard ratio: 5.56; 95% Confidence Intervals: 1.99–15.49; *p* = 0.001) [51]. The authors presented the minimum stent area (MSA) required in each segment of the LMCA to prevent significantly in-stent stenosis and MACEs. More specifically, the proposed MSA thresholds were 5.0 ${\mathrm{mm}}^{2}$ for the LCx ostium, 6.3 ${\mathrm{mm}}^{2}$ for the LAD ostium, 7.2 ${\mathrm{mm}}^{2}$ for the polygon of confluence, and 8.2 ${\mathrm{mm}}^{2}$ for the LMCA. These cutoffs also known as the “5-6-7-8 rule” concern Korean patients, whereas in Caucasians larger stent areas are needed, due to the greater body surface area. The prognostic role of MSA was validated by the recent EXCEL trial, which showed that the greater values of MSA are associated with less adverse events [54]. Notably, stent malposition was not correlated with more local or systemic complications, but further studies are required to confirm this finding [51].

Real-world practice has shown that performing IVUS in LMCA disease management is highly beneficial. To the best of our knowledge, Saleem *et al*. [65] have conducted the largest meta-analysis about the prognostic role of IVUS on LMCA disease management. A total of 12 studies (2 randomized-controlled trials [RCTs] and 10 observational studies) were analyzed, resulting in considerable results; all-cause mortality (OR: 0.57, 95% CI: 0.46–0.70, *p *< 0.00001), cardiovascular mortality (OR: 0.37, 95% CI: 0.26–0.54, *p *< 0.00001), left-main revascularization (OR: 0.63, 95% CI: 0.45–0.89, *p* = 0.009), and myocardial infarction (OR: 0.80, 95% CI: 0.66–0.97, *p* = 0.02) were significantly lower in the IVUS-guided arm [65]. Moreover, Ye and colleagues [66] included ten studies totaling more than 6400 patients, concluding that significant benefit from IVUS-guided PCI exists. More specifically, IVUS-guided PCI was linked to a significantly lower risk of all-cause death (risk ratio (RR): 0.60; 95% CI: 0.47–0.75; *p *< 0.001), cardiac death (RR: 0.47; 95% CI: 0.33–0.66; *p *< 0.001), target lesion revascularization (TLR) (RR: 0.43; 95% CI: 0.25–0.73; *p* = 0.002), and stent thrombosis (RR: 0.28; 95% CI: 0.12–0.67; *p* = 0.004) [66]. These findings were confirmed by other smaller meta-analyses [67, 68]. These meta-analyses retrieved data from numerous observational studies and RCTs, which are reviewed in Table 2 (Ref. [69, 70, 71, 72, 73, 74, 75, 76, 77, 78, 79]).

**Table 2.** **Main studies comparing IVUS-guided and ICA-guided PCI in LMCA disease**.

First author	Year of publication	Country	Design	Centers	Number of patients	Follow-up	Highlights
Park *et al*. [69]	2009	Korea	Observational registry	Multicenter	756/219	3	↓ mortality rate in IVUS-guided arm
							∼ MI and TVF
de la Torre Hernandez *et al*. [70]	2014	Spain	Pooled analysis of observational registries	Multicenter	505/1165	3	↓ composite endpoint (cardiac death, MI or TLR) in IVUS-guided arm
							↓ all-cause mortality in IVUS-guided arm
							↓ stent thrombosis in IVUS-guided arm
Gao *et al*. [71]	2014	China	Observational	Single Center	337/679	1	↓ composite endpoint (cardiac death, MI or TLR) in IVUS-guided arm
Tan *et al*. [72]	2015	Saudi Arabia	Randomized	Single Center	61/62	2	∼ MI and death
							↓ TVF
Kim *et al*. [73]	2017	Korea	Observational	Single Center	122/74	3	∼ all-cause, cardiovascular mortality and MI
Andell *et al*. [74]	2017	Sweden	Observational registry	Multicenter	621/1847	10	↓ Composite endpoint (all-cause death, restenosis, or definite stent thrombosis) in IVUS-guided arm
							↓ all-cause death in IVUS-guided arm
Tian *et al*. [75]	2017	China	Observational	Single Center	713/1186	3	↓ all-cause mortality in IVUS-guided arm
							↓ MI in IVUS-guided arm
Liu *et al*. [76]	2019	China	Randomized	Single Center	167/169	1	↓ composite endpoint (cardiac death, MI or TVF) in IVUS-guided arm
							∼ stent thrombosis
Choi *et al*. [77]	2019	Korea	Observational	Single Center	453/251	5	↓ cardiac death and adverse events in IVUS-guided arm
Kinnaird *et al*. [78]	2020	United Kingdom	Observational Registry	Multicenter	5056/6208	1	↓ composite endpoint (death, stroke or MI) in IVUS-guided arm
							↑ one- and twelve months survival in IVUS-guided arm
de la Torre Hernandez *et al*. [79]	2020	Spain	Observational Registry	Multicenter	124/124	1	↓ composite endpoint (cardiac death, LMCA-related MI and LMCA revascularization)

ICA, Invasive Coronary Angiography; IVUS, Intravascular Ultrasound Imaging; MI, Myocardial Infraction; LMCA, Left Main Coronary Artery; PCI, Percutaneous Coronary Interventions; TLR, Target Lesion Revascularization; TVF, Target Vessel Failure.

### 3.3 Optical Coherence Tomography

OCT is a modern imaging modality used in several medical fields, such as ophthalmology and cardiology [80, 81, 82]. OCT uses coherent infrared light to depict the microstructure within coronary arteries. The technology of OCT provides better resolution than IVUS; however, the penetrating imaging depth into the arterial wall is significantly smaller [83]. Similar to IVUS catheters, OCT catheters contain an OCT head at the distal tip of the catheter. During the examination, automatic pullback and rotation of the catheter creates cross-sectional views of the coronary arteries. Contrast medium or other solutions are necessary, because blood reduces the quality of the OCT images [84].

During OCT imaging, normal coronary arteries are depicted as circular structures with three layers: the inner layer represents the internal elastic membrane, the middle, dark layer corresponds to the median layer, and the outer layer is the external elastic lamina [85, 86].

Similar to IVUS, OCT should be performed before angioplasty for the evaluation of plaque composition and extent, the identification of the lesion’s anatomical characteristics, and the choice of the appropriate stent size. Moreover, OCT has been deemed reliable for detecting vulnerable plaque. According to the existing knowledge, atherosclerotic plaques with specific morphological characteristics are more prone to rupture and promote thrombosis, which subsequently leads to the clinical manifestation of ACS. Due to its high resolution, OCT can detect timely and precisely these characteristics, such as the thickness of the overlying fibrous plaque, and contribute to improved invasive and pharmaceutical management [87]. OCT imaging post PCI is of paramount importance for optimal stent deployment and timely recognition of post-procedural complications [87, 88].

OCT is less studied than IVUS in LMCA disease. The ROCK I trial compared OCT-guided LMCA PCI with standard (angiographic ± IVUS) PCI, retrospectively. Although no clinical difference was observed between the two groups, late lumen loss tended to be lower in the OCT arm and was significantly reduced in the distal part of the main vessel. Moreover, OCT-guidance contributed to the detection of cases with underexpansion and malposition of stents [89].

Roule and colleagues [90] supported that more than 90% of the quadrants of the LMCA were adequately assessable by newer-generation OCT, while most artifacts were located at the proximal part of the LM. A study by Burzotta *et al*. [91] confirmed that the OCT evaluation of the distal LM is more accurate and efficient, compared to the more proximal segments of the LMCA, where the diagnostic ability of OCT is poor.

Bouki *et al*. [92] confirmed the inability of OCT to evaluate proximal lesions, as only half of the plaques located in the proximal LMCA could be analyzed. Moreover, they claimed that the OCT-derived MLA of ≤5.38 ${\mathrm{mm}}^{2}$ accurately predicts the functional severity of LMCA disease. Nevertheless, further studies with OCT should be conducted for defining OCT-derived MLA criteria and not extrapolating data by IVUS, due to the existing discrepancy between the two methods [48].

The first prospective trial assessing the role of OCT in LMCA PCI, according to a prespecified protocol, is LEMON. Sufficient stent expansion was noticed in 86%, edge dissection in 30%, and residual strut malapposition in 24% of the patients. Interestingly, approximately one in four operators (26%) changed their therapeutic strategy because of the post-PCI OCT, despite the sufficient angiographic results [93].

The presence of calcium in LMCA lesions has been associated with higher rates of stent thrombosis, target vessel failure, and myocardial infraction [94, 95, 96]. Although IVUS can provide decent information about calcified lesions, OCT is more precise in estimating calcium thickness and whether it can affect stent expansion. It has been reported that patients with calcium deposit with a maximum angle greater than 180°, length more than 5 mm, and maximum thickness higher than 0.5 mm were at risk of stent underexpansion and subsequent stent stenosis [97]. In such cases, the interventional cardiologists could perform special techniques, such as rotational atherectomy, balloon dilation, or lithotripsy, in order to appropriately modify the plaques.

OCT is useful for evaluating stent failure. Specifically, OCT could provide critical information regarding the underlying mechanism of failure, such as neoatherosclerosis, neointimal hyperplasia, stent thrombosis, underexpansion, or fracture. Thus, the appropriate treatment could be chosen and preventive measures for repetitive stent failure could be applied [98]. However, OCT usage demands higher dose of contrast agent and could be a major problem in patients with renal impairment. Taking into consideration that LMCA and PCI requires multiple periprocedural manipulations and increased contrast agent dose, OCT should be performed with caution in such patients. In this regard, low- or no-contrast administration during OCT has been investigated [99].

## 4. Choosing the Optimal Imaging Modality for LMCA Disease

Owing to all the aforementioned imaging modalities, modern interventional cardiologists can handle LMCA disease more efficiently, compared to a decade ago. First, CCTA can rule out moderate or severe LMCA disease noninvasively. ICA remains the gold standard for evaluating CAD; however, every intervention performed in LMCA should be assisted by IVUS or OCT. It is evident that OCT- or IVUS-guided PCI is superior to angiographic-guided angioplasty in almost every case of LMCA stenting [100].

A few studies have directly compared IVUS and OCT in the management of left main disease. Fujino and colleagues [101] were the first to directly compare newer-generation OCT and IVUS in a prospective cohort, by performing both OCT and IVUS pre- and post-PCI in 35 patients. The two techniques achieved comparable results in measuring mean lumen and stent areas (11.24 ± 2.66 vs. 10.85 ± 2.47 ${\mathrm{mm}}^{2}$, *p* = 0.13 and 10.44 ± 2.33 vs. 10.49 ± 2.32 ${\mathrm{mm}}^{2}$, *p* = 0.82, respectively); OCT was superior in detecting stent malapposition and distal edge dissections. However, IVUS produced more comprehensive and qualitative images, in total, mainly in the ostial LMCA [101].

A recent study compared three-dimensional OCT versus IVUS in LMCA disease stenting. In more than 300 patients included, the cumulative rate of the primary endpoint (a composite of cardiac death, myocardial infarction, and target lesion revascularization) was comparable between the two, both before and after propensity score adjustment (7.0% vs. 7.4%, *p* = 0.98 and 2.6% vs. 7.3%, *p* = 0.18). Thus, three-dimensional OCT- and IVUS-guided angioplasty for LMCA disease were equally feasible and safe [102].

The ROCK cohort II study was a multicenter, investigator-initiated, retrospective study which compared the performance of intravascular imaging modalities and angiography in patients undergoing distal-LMCA angioplasty. The authors did not identify any differences between OCT and IVUS with regards to the target-lesion failure [103].

Generally, IVUS has been studied more extensively in LM disease, resulting in greater familiarization and clinical experience. Due to the higher penetration depth, IVUS can image all the arterial layers and assess coronary artery remodeling. Undoubtedly, IVUS outbalances OCT in the imaging of ostial disease; arteries with large diameter (especially larger than 4 mm) present higher risk for blood contamination, which negatively affects OCT image quality. While OCT is less studied in LMCA disease, it provides significantly higher resolution and depicts the details of plaques and stents more accurately. As a result, OCT remains superior regarding stent underexpansion, dissection or malapposition, as well as in thrombi imaging.

Calcified lesions are a “grey-zone”: for intravascular imaging. OCT provides more information about calcium depth and IVUS can adequately visualize only the superficial calcium layer.

Moreover, OCT requires more contrast agent during the procedure to achieve better image quality. Thus, IVUS should probably be preferred in patients with renal impairment.

## 5. Future Perspectives

During the two last decades, intravascular imaging modalities have developed remarkably, but further steps are required for the better depiction, evaluation, and management of LMCA lesions. Regarding OCT, the lack of a well-established threshold for MLA remains an important limitation. Ongoing studies, such as OCTOBER (NCT03171311, clinicaltrials.gov) and ILUMUEN IV (NCT03507777, clinicaltrials.gov) should set the cutoff for OCT and investigate its role in clinical practice more comprehensively.

The progress in technology and biophysics will significantly contribute to the evolution of IVUS. The first devices combining IVUS with near-infrared spectroscopy (NIRS) have been recently released. Although the existing literature is limited, integrated IVUS-NIRS systems are thought to provide more detailed information regarding atherosclerotic plaque morphology and erosion risk [104, 105]. However, no study on the applications of IVUS-NIRS in LMCA disease has been conducted yet. The combination of IVUS with OCT may attract attention in the near future. Simultaneous performance of OCT and IVUS examination as co-registration has been applied in some catheterization laboratories [106]. Moreover, IVUS and OCT were integrated into a hybrid, single catheter system. The novel, hybrid OCT-IVUS catheter aims to achieve optimal depiction of lesions in the coronary arteries [107]. Invasive imaging could play a role in the management of less frequent causes of ACS, such spontaneous coronary artery dissection (SCAD). Because SCAD is poorly described and extremely rare in LMCA, further studies are required in order to identify the real benefit of using intravascular imaging in these cases [108, 109].

Nevertheless, intravascular imaging cannot be considered as panacea, because it might not be suggestive in several cases. On the other hand, the assessment of coronary physiology using fractional flow reserve (FFR) could be assistive [110, 111]. The combination of these methods could contribute to the optimal management of such patients; nevertheless, further studies are required to confirm this claim, especially as far as LMCA disease is concerned [35, 112]. For ‘grey-zone’ lesions, in which the optimal management remains unclear, the IVUS ‘virtual histology’ option could be helpful. This is an IVUS-based post-processing modality for spectral interpretation of the primary raw backscattered radiofrequency. After the processing of black and white images, the tissues are color-coded as four major components; dense calcium (white), necrotic core (red), fibro-fatty (light green), and fibrous tissue (dark green) [113, 114].

Newer technologies will allow three dimensional (3D)-reconstruction to achieve a more realistic depiction of the anatomy and morphology of the lesions [115, 116]. Moreover, artificial intelligence and deep learning systems will expand intravascular imaging capabilities [117, 118].

## 6. Conclusions

In conclusion, imaging in LMCA disease is crucial for achieving optimal results. Especially in patients undergoing PCI, intravascular imaging is considered as mandatory before, during, and after angioplasty. IVUS has been performed and studied more extensively, but OCT provides special advantages. Undoubtedly, the progress in technology will evolve intravascular imaging modalities, increasing their precision in challenging cases, such as patients with LMCA disease.
